# Genetic diversity of *Plasmodium vivax* populations from the China–Myanmar border identified by genotyping merozoite surface protein markers

**DOI:** 10.1186/s41182-022-00492-7

**Published:** 2023-01-11

**Authors:** Xun Wang, Yao Bai, Zheng Xiang, Weilin Zeng, Yanrui Wu, Hui Zhao, Wei Zhao, Xi Chen, Mengxi Duan, Xiaosong Li, Wenya Zhu, Kemin Sun, Yiman Wu, Yanmei Zhang, Xiaomei Li, Benjamin M. Rosenthal, Liwang Cui, Zhaoqing Yang

**Affiliations:** 1 Department of Pathogen Biology and Immunology, Kunming Medical University, Kunming, 650500 Yunnan China; 2 Department of Cell Biology and Genetics, Kunming Medical University, Kunming, China; 3 Faculty of Public Health, Kunming Medical University, Kunming, Yunnan Province China; 4 Animal Parasitic Disease Laboratory, Agricultural Research Service, US Department of Agriculture, Beltsville, MD USA; 5 Department of Internal Medicine, Morsani College of Medicine, University of South Florida, Tampa, FL 33612 USA

**Keywords:** *Plasmodium vivax*, Genotyping, Merozoite surface protein, Mixed-strain infection, Multiplicity of infection, Population differentiation

## Abstract

**Background:**

Parasite diversity and population structure influence malaria control measures. Malaria transmission at international borders affects indigenous residents and migrants, defying management efforts and resulting in malaria re-introduction. Here we aimed to determine the extent and distribution of genetic variations in *Plasmodium vivax* populations and the complexity of infections along the China–Myanmar border.

**Methods:**

We collected clinical *P. vivax* samples from local and migrant malaria patients from Laiza and Myitsone, Kachin State, Myanmar, respectively. We characterized the polymorphisms in two *P. vivax* merozoite surface protein markers, *Pvmsp-3α* and *Pvmsp-3β*, by PCR-restriction fragment length polymorphism (PCR–RFLP) analysis. We sought to determine whether these genetic markers could differentiate these two neighboring parasite populations.

**Results:**

PCR revealed three major size variants for *Pvmsp-3α* and four for *Pvmsp-3β* among the 370 and 378 samples, respectively. PCR–RFLP resolved 26 fragment-size alleles by digesting *Pvmsp-3α* with *Alu* I and *Hha* I and 28 alleles by digesting *Pvmsp-3β* with *Pst* I. PCR–RFLP analysis of *Pvmsp-3α* found that infections in migrant laborers from Myitsone bore more alleles than did infections in residents of Laiza, while such difference was not evident from genotyping *Pvmsp-3β*. Infections originating from these two places contained distinct but overlapping subpopulations of *P. vivax.* Infections from Myitsone had a higher multiplicity of infection as judged by the size of the *Pvmsp-3α* amplicons and alleles after *Alu* I/*Hha* I digestion.

**Conclusions:**

Migrant laborers from Myitsone and indigenous residents from Laiza harbored overlapping but genetically distinct *P. vivax* parasite populations. The results suggested a more diverse *P. vivax* population in Myitsone than in the border town of Laiza. PCR–RFLP of *Pvmsp-3α* offers a convenient method to determine the complexity of *P. vivax* infections and differentiate parasite populations.

**Supplementary Information:**

The online version contains supplementary material available at 10.1186/s41182-022-00492-7.

## Background

Malaria has been a major threat to public health and economic development [1]. With an estimated 13.8 million victims each year, *Plasmodium vivax* is widespread, occurring in endemic areas of Asia, Oceania, Central and South America, the Middle East, and West Africa [2]. Increasing imported malaria cases in mainland China often originate in Myanmar, which has the highest malaria burden in the Greater Mekong Subregion (GMS) [3]. In southwestern China, especially in Yunnan Province, malaria is particularly prevalent among border crossers, and ethnic minority groups in the border areas of Myanmar–Yunnan are at elevated risk. As the GMS aims to eliminate malaria by 2030, it is critical to understand the parasite populations and transmission dynamics in the border areas.

Genetic markers have been used to determine the diversity of parasite populations, spatial and temporal transmission dynamics, and complexity of infections. Molecular markers are also used to quantify the intensity of infections and determine the number of parasites harbored by an individual (the multiplicity of infection, or MOI). Many merozoite surface proteins, such as *msp1*, *msp2*, and *glurp* in *Plasmodium falciparum* [4], and *msp1* and *msp3α/β* in *P. vivax* [6], have been used extensively for parasite genotyping since they display significant variations. While sequencing of regions of these genes can reveal much higher genetic diversity, another method that is often used for genotyping these markers is polymerase chain reaction-restriction fragment length polymorphism (PCR–RFLP), which offers the advantages of high sensitivity, convenience, and low cost [6].

The *Pvmsp-3α* and *Pvmsp-3β* genes encode merozoite surface proteins with an alanine-rich central domain, which strongly predicts coil-like tertiary structure [8]. The central domain is prone to large insertion/deletion mutations, thus displaying substantial length variations [10]. The *Pvmsp-3α* length polymorphism was first identified in parasites from Asia, South America, and the South Pacific region, with four major amplified fragment types reported [6]. Similarly, length variations in *Pvmsp-3β* allow the differentiation of *P. vivax* from different geographical areas [12]. When combined, two markers provide increasing power to differentiate parasite isolates [13]. For example, *Pvmsp-3α* and *Pvmsp-3β* have been used to study the genetic diversity of *P. vivax* from Pakistan [7] and Kachin State, Myanmar [14].

The continued transmission of *P. vivax* in the border region of Myanmar and its contribution to imported malaria to China prompted us to investigate the changes in the parasite population and the spatio-temporal transmission dynamics in the border region [15]. Specifically, we sought to characterize the polymorphisms of *Pvmsp-3α* and *Pvmsp-3β* from two *P. vivax* populations along the China–Myanmar border to see if they can be used to differentiate these parasite populations and trace the parasite origins*.* Our study identified that migrant workers, who had acquired *P. vivax* infection from Myitsone while constructing the Myitsone Dam [17], harbored more genetically diverse *P. vivax* than the local malaria patients from Laiza.

## Materials and methods

### Sample collection, malaria diagnosis and DNA extraction

**Fig. 1:**
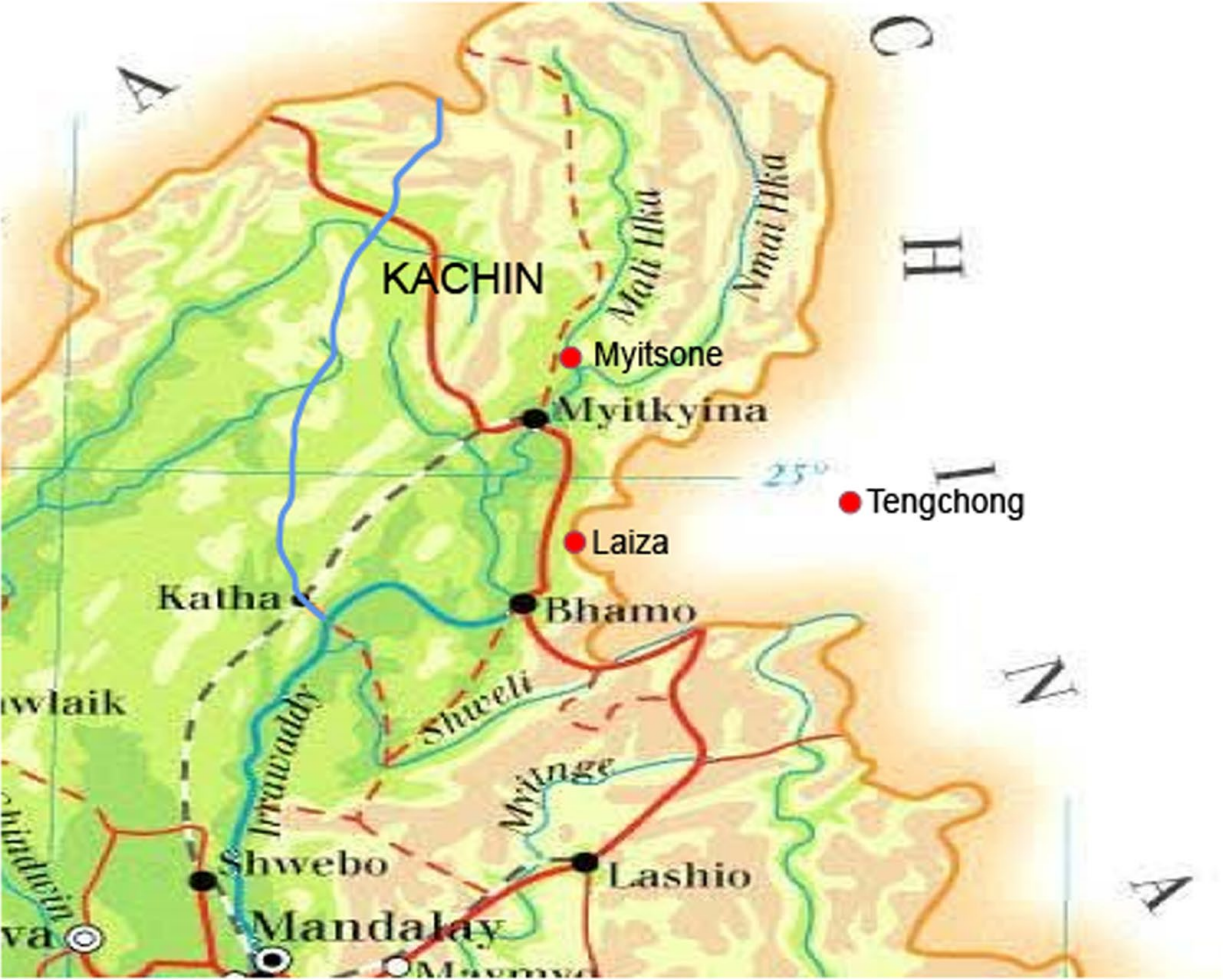
*Plasmodium vivax* sampling sites

In this study, 442 samples of *P. vivax* were analyzed from symptomatic patients in hospitals or malaria clinics in two regions of the China–Myanmar border in 2012–2015. Among them, 230 samples were collected from 13 clinics around Laiza township, Kachin State, Myanmar (Fig. 1), during the high malaria season (June–September) in 2012–2013 (43 samples) and 2015 (187 samples). In 2013, 192 samples were collected from Chinese migrant laborers-patients returning from Myitsone, Kachin, through the immigration control checkpoints of Tengchong (Yunnan Province, China) (Fig. 1). These two areas, Laiza and Myitsone, represent regional hotspots for the transmission of *P. vivax* [18]. All patients had uncomplicated malaria, with a parasite density of 752–3579/μl. Each patient voluntarily signed an informed consent form. This study was reviewed and approved by the Institutional Review Board of Kunming Medical University.

Malaria infection was confirmed by microscopy of Giemsa-stained thick and thin blood films. Two five-milliliters of venous blood were drawn from each patient and stored at − 20 °C until use. Genomic DNA was extracted from 100 μl of whole blood using the High Pure PCR Template Preparation Kit (Roche, Germany).

### Amplification of *Pvmsp-3α *and *Pvmsp-3β* genes

Nested PCR and genotyping of *Pvmsp-3α* and *Pvmsp-3β* used previously described methods [12] with modifications. Oligonucleotide primers and cycling conditions are listed in Additional file 1: Table S1 and Additional file 2: Fig. S1. For the first round of amplification, 1.0 μl DNA was added to a reaction of 25 μl, including 1.25 U TaKaRa Taq polymerase, 3 mM MgCl_2_, 0.4 mM dNTPs, distilled H_2_O, and 0.2 μl of each primer (10 pM). For nested PCR, 2.0 μl of the first amplification product was used as the template and added to a total volume of 50 μl of the reaction system, including distilled H_2_O, primers and Premix Taq™ (TaKaRa Taco™ version 2.0 plus dye). The PCR products were resolved on 0.8% agarose gel, and their sizes were determined using a 200 bp DNA ladder (Dye Plus; TaKaRa, Japan).

### PCR–RFLP genotyping of *Pvmsp-3α *and *Pvmsp-3β*

Allelic polymorphism of the *Pvmsp-3a* and *Pvmsp-3β* genes was assessed using established PCR–RFLP techniques. Briefly, 3 μl of each PCR product were digested with restriction enzymes for more than 5 h. *Pvmsp-3a* amplicons were digested in 10 μl of reactions with 1 μl of *Alu* I (enzyme cutting site: 5′-AGC|T-3′) or *Hha* I (enzyme cutting site: 5′-GCG|C-3′). *Pvmsp-3β* was digested with 1.5 μl of *Pst* I (enzyme cutting site: 5′-TTA|TAA-3′) in 11 μl reactions (TaKaRa, Japan). Restriction fragments were separated and visualized in 1.8% agarose gels.

### Mixed-strain infections and MOI

Mixed-strain infections were inferred when PCR products of more than one size appeared in the initial amplification [6] or when the summed size of the DNA fragments resulting from RFLP restriction exceeded the size of the uncut PCR product [21]. Alleles were identified based on unique restriction banding patterns. Alleles were considered the same if the restriction banding patterns were estimated to be within 20 bp [22]. MOI denotes the number of distinct parasite genotypes in the same patient [24]. When the total fragment length generated by a single sample exceeded the mean value of the uncut band, but was less than twice the mean value of the uncut band, we judged the sample to constitute mixed infections of two subtypes. We estimated MOI for each patient to compare residents of Laiza with migrant laborers using *Pvmsp-3α* and *Pvmsp-3β* genotypes.

### Statistical analysis

The Chi-square (χ^2^) test was used to compare the frequencies of RFLP alleles among the populations using the Prism GraphPad 6. Fisher’s exact test was used to compare the differences between two populations in individual alleles. *T*-test was used to compare the infection multiplicity between two populations. *P* values of < 0.05 were considered significant.

## Results

### Genotyping *Pvmsp-3α* and *Pvmsp-3β* genes by PCR

**Fig. 2:**
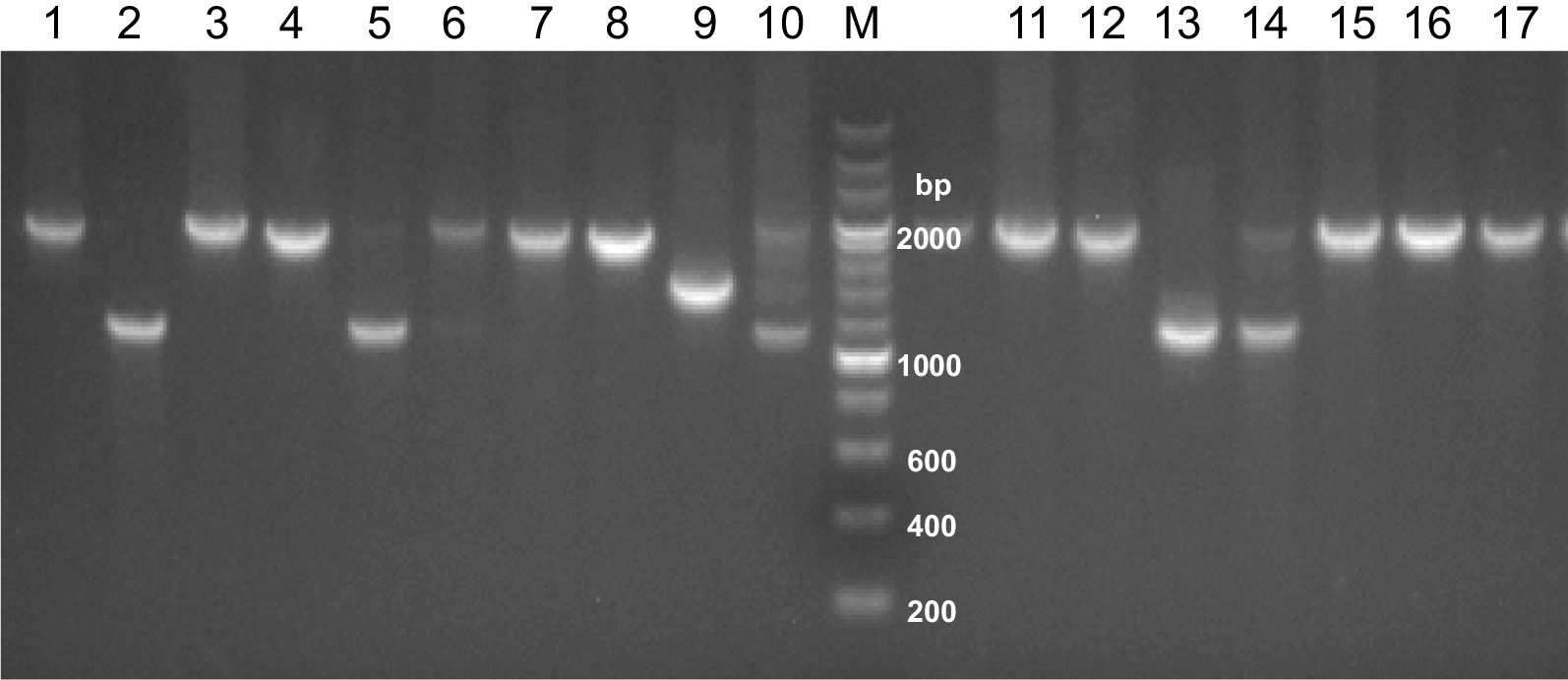
Examples of major fragment lengths of the *Pvmsp-3α* amplicon. M, DNA marker in bp. Type A: lanes 1, 3, 4, 7, 8, 11, 12, 15, 16, 17; Type B: lane 9; Type C: lanes 2, 5, 12; mixed infections: lanes 10, 14

**Fig. 3:**
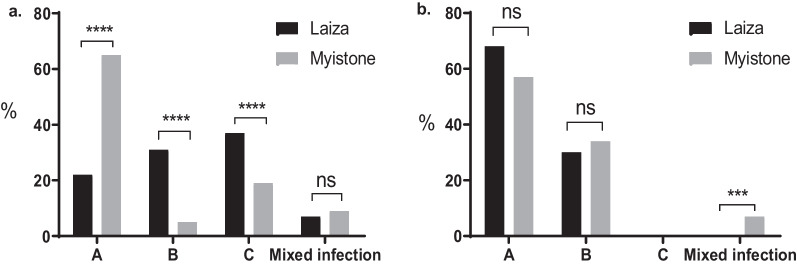
Proportions of each amplicon type of *Pvmsp-3α* (**a**) and *Pvmsp-3β* (**b**) genes in Laiza and Myitsone regions. ***P < 0.05; ****P < 0.0001 (Fisher’s exact test)

For *Pvmsp-3a,* we successfully genotyped 227 of 230 (98.7%) samples from Laiza and 178 of 192 (92.7%) Myitsone samples from Chinese migrant patients. Three different target bands (types A, B, and C, measuring 1900–2000, 1400–1500, and 1100–1300 bp, respectively) were amplified (Fig. 2). Among the samples from Laiza, those with multiple PCR bands occurred in 7.9% (18/227). Single infections of types A, B, and C occurred at frequencies of 22.9, 31.1, and 37.9%, respectively. In comparison, 9.5% (17/178) of the Myitsone samples from the migrants yielded multiple bands. Single infections of type A, B, and C occurred at 65.2, 5.6, and 19.7%, respectively (Fig. 3a). Thus, the three allele types occurred with approximate parity in Laiza, but type A predominated in the Myitsone population from migrant workers. Mixed infections diagnosed by *Pvmsp-3a* occurred at a similar frequency (*P* < 0.05).

**Fig. 4:**
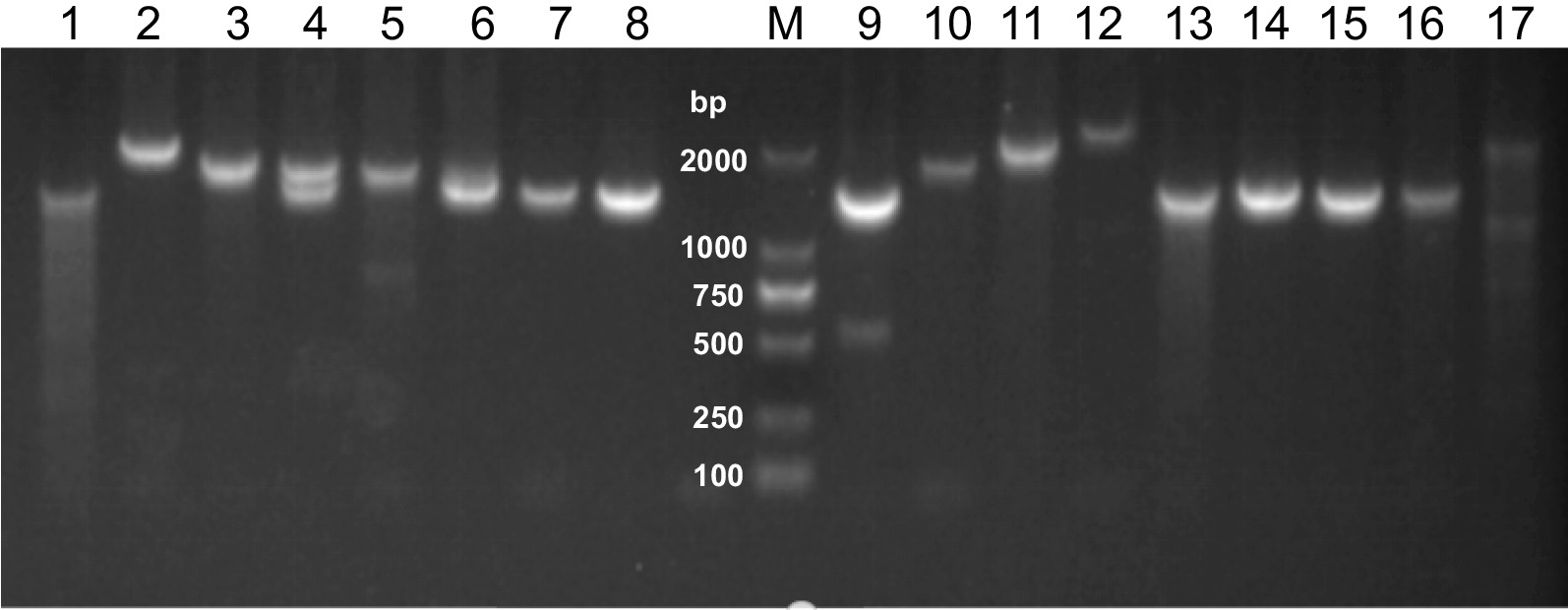
Examples of major fragment lengths of the *Pvmsp-3β* amplicon. M, DNA marker in bp. Type A: lanes 2, 10, 11, 12; Type B, lanes 1, 3, 5, 6, 7, 8, 13, 14, 15; mixed infection: lanes 4, 9 (Type D mixed infection), 17 (Type C mixed infection)

*Pvmsp-3β* was successfully amplified in 220 of 230 (95.7%) Laiza samples and 173 of 192 (90.1%) Myitsone samples. The *Pvmsp-3β* PCR products had four amplicon length types (A, B, C, and D), corresponding to 1700–2200, 1400–1500, 1100–1300, and 600–800 bp, respectively (Fig. 4). Among the samples from Laiza, multiple band samples accounted for 0.9% (2/220), while single-band samples with types A, B, and D occurred at 68.6, 30.0, 0.5%, respectively. Types A and B were predominant, accounting for over 98.6% of the samples. Only one D-type allele was identified in a single patient, while Type C appeared only in mixed infections. In comparison, the Myitsone samples yielding multiple *Pvmsp-3β* PCR bands occurred at 7.5% (13/173). Notably, single-band samples were only present in two types; Type A occurred in 57.8% and type B in 34.7%. Types C and D appeared only in mixed infections (Fig. 3b). Thus, Types A and B predominated at comparable frequencies in the two regions. Significantly more mixed infections were detected among migrant workers than residents of Laiza by genotyping *Pvmsp-3β* (*P* > 0.05).

### RFLP results of *Pvmsp-3α* gene

**Fig. 5:**
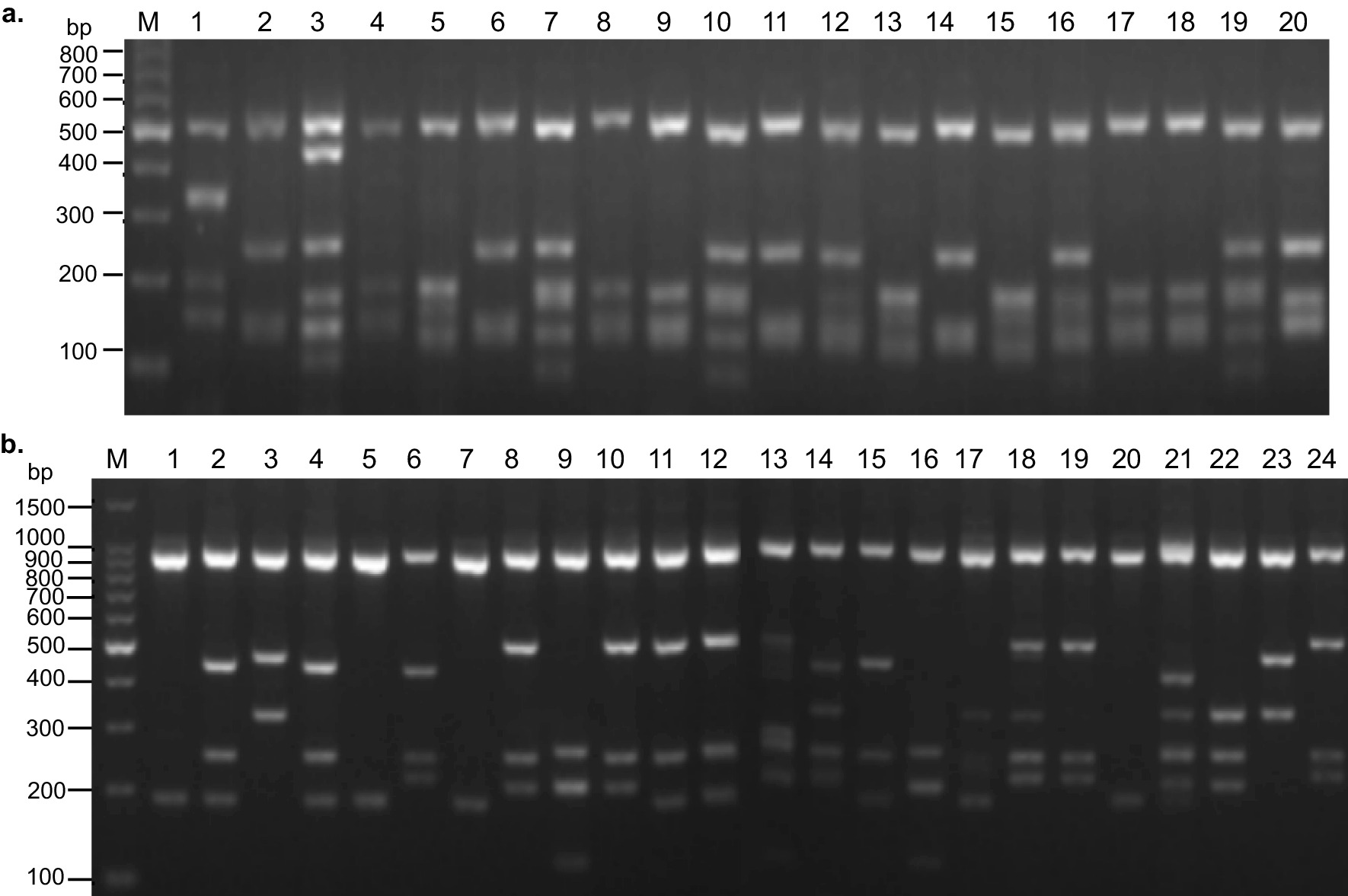
Major RFLP alleles of the *Pvmsp-3α* amplicons digested with *Alu* I (**a**) and *Hha* I (**b**). Lane M, DNA marker in bp. **a** Lane 1—B3; lanes 2, 6, 11, 14, 20—C1; lane 3—A7; lanes 4, 8, 9, 17, 18—A5; lanes 5, 13, 15—B4; lanes 7, 10, 19—A1; lanes 12, 16, 20—A4. **b** Lanes 1, 5, 7, 20—C1; lanes 2, 4, 11, 12, 15—A1; lanes 3, 23—A2; lanes 6, 8, 10, 19, 24—A3; lanes 9, 16—A4; lanes 14, 21—A13; lanes 17, 22—A8; lane 18—A14

Figure 5a depicts the band patterns resulting from *Alu* I digestion of the amplified *Pvmsp-3α* gene. A total of 16 isoforms of Type A, 7 isoforms of Type B, and 3 isoforms of Type C were apparent in the two regions. Each region had 19 alleles, but with different allele types or frequencies. In the Laiza samples, 10 A, 6 B, and 3 C isoforms occurred, while in Myitsone samples, 12 A, 4 B, and 3 C isoforms occurred (Table 1). No significant difference was observed in the frequency distribution of isoforms across the two sampling locales (χ^2^ = 0.582, *P* = 0.748). However, the two sample populations had significantly different distributions of Type A alleles of *Pvmsp-3a*. The same is true for the alleles of Type B and C (Table 1).

**Table 1:** *Pvmsp-3α Alu* I PCR–RFLP allele types and frequencies in two *Plasmodium vivax* populations

Allele (PCR–RFLP)	Frequency of allelic variants [*n* (%)]		*P* values (χ^2^ test)
	Laiza (*N* = 209)	Myitsone (*N* = 161)	
A1	2 (1.0)	19 (11.8)	χ^2^ = 9.035, *P* < 0.0001
A2	10 (4.8)	7 (4.3)	
A3	10 (4.8)	5 (3.1)	
A4	16 (7.7)	31 (19.3)	
A5	1 (0.5)	19 (11.8)	
A6	5 (2.2)	0	
A7	1 (0.5)	0	
A8	2 (1.0)	0	
A9	1 (0.5)	0	
A10	4 (1.9)	4 (2.5)	
A11	0	1 (0.6)	
A12	0	1 (0.6)	
A13	0	5 (3.1)	
A14	0	8 (5)	
A15	0	11 (6.8)	
A16	0	5 (3.1)	
B1	32 (15.3)	4 (2.5)	χ^2^ = 6.402, *P* < 0.0001
B2	4 (1.9)	0	
B3	6 (2.9)	0	
B4	24 (11.5)	2 (1.2)	
B5	1 (0.5)	3 (1.9)	
B6	4 (1.9)	0	
B7	0	1 (0.6)	
C1	59 (28.2)	18 (11.3)	χ^2^ = 3.946, *P* < 0.0001
C2	23 (11.5)	16 (9.9)	
C3	3 (1.4)	1 (0.6)	
Total # of alleles	19	19	

Figure 5b depicts the results of *Hha* I digestion of *Pvmsp-3α*, revealing 16 Type A, 4 B, and 6 C isoforms. Laiza parasites had 17 isoforms (9 A, 3 B, and 5 C isoforms), compared to 23 isoforms in Myitsone (15 A, 2 B, and 6 C isoforms) (Table 3). The distribution of the *Pvmsp-3α Hha* I isoforms did not differ significantly between the two populations (χ^2^ = 0.911, *P* = 0.634). However, as described for *Alu* I digestion, the frequency distribution of these alleles was significantly different between the two populations (Table 2).

**Table 2:** *Pvmsp-3α Hha* I PCR–RFLP allele types and frequencies in two *P. vivax* populations

Allele (PCR–RFLP)	Frequency of allelic variants [*n* (%)]		*P* values (χ^2^ test)
	Laiza (*N* = 209)	Myitsone (*N* = 161)	
HA1	3 (1.4)	1 (0.6)	χ^2^ = 9.035, *P* < 0.0001
HA2	1 (0.5)	2 (1.2)	
HA3	2 (1.0)	7 (4.3)	
HA4	6 (2.9)	4 (2.5)	
HA5	8 (3.8)	4 (2.5)	
HA6	17 (8.1)	11 (6.8)	
HA7	1 (0.5)	0	
HA8	4 (1.9)	17 (10.6)	
HA9	10 (4.8)	13 (8.1)	
HA10	0	7 (4.3)	
HA11	0	5 (3.3)	
HA12	0	1 (0.6)	
HA13	0	3 (1.9)	
HA14	0	2 (1.2)	
HA15	0	35 (21.7)	
HA16	0	4 (2.5)	
HB1	5 (2.4)	0	χ^2^ = 6.402, *P* < 0.0001
HB2	39 (19.1)	7 (4.3)	
HB3	26 (12.4)	0	
HB4	0	3 (1.9)	
HC1	2 (1.0)	1 (0.6)	χ^2^ = 3.946, *P* < 0.0001
HC2	1 (0.5)	1 (0.6)	
HC3	7 (3.3)	13 (8.1)	
HC4	75 (35.9)	16 (9.9)	
HC5	1 (0.5)	1 (0.6)	
HC6	0	3 (1.9)	
Total # of alleles	17	23	

### RFLP results of *Pvmsp-3β* gene

**Fig. 6:**
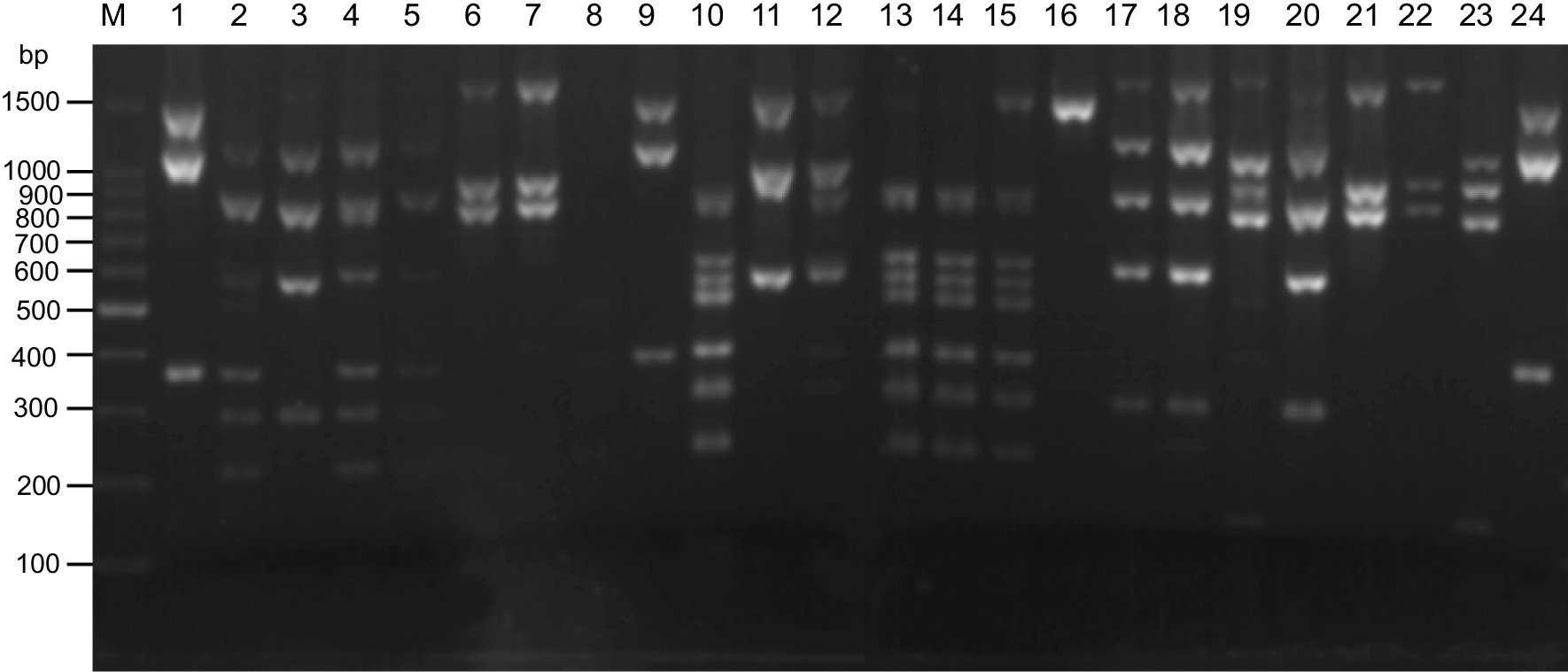
Major RFLP alleles of the *Pvmsp-3β* amplicons digested with *Pst* I. M, DNA marker in bp. Lanes 1, 9, 24—A5; lanes 2, 4, 5—A4; lanes 3, 17, 18, 20—A1; lanes 6, 7, 21, 22—A14; lanes 10, 13, 14, 15—B2; lanes 11, 12—A8; lane 16—B9; lanes 19, 23—B3

*Pst* I digestion of the *Pvmsp-3β* amplicon identified 14 Type A and 13 Type B isoforms (Fig. 6). The two populations harbored distinct distributions of these alleles (Table 3). The Laiza samples had 26 isoforms (14 A, 11 B, and 1 D); alleles A4, A5, A7, A9, A11, and B9 were most abundant, accounting for 52.3% of all samples. In the Myitsone population, fewer isoforms were observed (10 A and 9 B). Isoforms A11, A12, and B13 comprised 59% of the samples. The allele frequency distribution, however, did not differ significantly between the two populations (χ^2^ = 0.0065, *P* = 0.9357). Similarly, examining the distribution of A and B alleles separately failed to identify a significant difference between the two parasite populations (Table 3).

**Table 3:** *Pvmsp-3β Pst* I PCR–RFLP allele types and frequencies in two *P. vivax* populations

Allele (PCR–RFLP)	Frequency of allelic variants [*n* (%)]		*P* values (χ^2^ test)
	Laiza (*N* = 218)	Myitsone (*N* = 160)	
PA1	9 (4.3)	2 (1.2)	χ^2^ = 1.376, *P* = 0.1688, *P* > 0.05
PA2	5 (2.3)	0	
PA3	5 (2.3)	0	
PA4	26 (11.5)	9 (5.6)	
PA5	15 (6.9)	6 (3.8)	
PA6	8 (3.7)	2 (1.3)	
PA7	17 (7.8)	13 (8.1)	
PA8	5 (2.3)	0	
PA9	15 (6.9)	7 (4.4)	
PA10	11 (5.0)	11 (6.8)	
PA11	28 (12.8)	22 (13.8)	
PA12	3 (1.4)	22 (13.8)	
PA13	1 (0.4)	0	
PA14	3 (1.4)	6 (3.8)	
PB1	9 (4.3)	0	χ^2^ = 1.427, *P* = 0.141, *P* > 0.05
PB2	5 (2.3)	0	
PB3	2 (0.9)	1 (0.6)	
PB4	6 (2.8)	6 (3.8)	
PB5	8 (3.7)	4 (2.5)	
PB6	5 (2.3)	0	
PB7	8 (3.7)	5 (3.1)	
PB8	6 (2.8)	0	
PB9	13 (6)	2 (1.2)	
PB10	2 (0.9)	1 (0.6)	
PB11	0	4 (2.5)	
PB12	0	12 (7.5)	
PB13	2 (0.9)	25 (15.6)	
PD	1 (0.4)	0	*P* = 0.391*
Total # of alleles	26	19	

*Fisher’s exact test

### Multiplicity of infection (MOI)

PCR–RFLP analyses revealed occasional occurrences of mixed genotype infections in both parasite populations. The MOI of the Laiza samples ranged from 1 to 3 and 1 to 4, as judged by *Pvmsp-3a* and *Pvmsp-3β,* respectively. The MOI in the Myitsone samples ranged from 1 to 3 and 1 to 5 using these genes. Specifically, based on *Alu* I digestion of *Pvmsp-3a,* the MOI was estimated at 1.125 ± 0.412 and 1.173 ± 0.545 for the Laiza and Myitsone parasites (Table 4), respectively (*P* > 0.05). *Hha* I digestion yielded estimates of MOI at 1.23 ± 0.517 and 1.397 ± 0.637 (Table 4), respectively, which differed significantly (*P* < 0.01). When restricting the analysis of MOI to adults 18 and older, we found that the MOI, estimated by *Hha* I digestion of *Pvmsp-3a*, was significantly lower among the Laiza residents (1.221 ± 0.5099) than migrant workers (1.368 ± 0.6116) (*P* < 0.05).

**Table 4:** Comparison of multiplicity of infection (mean ± standard deviation) between Laiza and Myitsone patients

Marker	Laiza	Myitsone	P values*
*Pvmsp-3a Alu* I	1.125 ± 0.412	1.173 ± 0.545	> 0.05
*Pvmsp-3a Hha* I	1.23 ± 0.517	1.397 ± 0.637	< 0.01
*Pvmsp-3β Pst* I	2.04 ± 0.754	1.936 ± 0.882	> 0.05

*The *t* test

*Pst* I digestion of *Pvmsp-3β* yielded statistically equivalent estimates of MOI in the two patient populations (2.04 ± 0.754 vs. 1.936 ± 0.882; *P* > 0.05) (Table 4). Although this amplification/digestion assay yielded larger estimates of MOI, it proved poorer in differentiating MOI among the two population samples. Restricting the analysis to adults yielded a similarly non-significant result (2.072 ± 0.793 vs. 1.897 ± 0.864, *P* > 0.05).

## Discussion

Parasite genetic diversity illuminates the dynamics of malaria transmission and the fate of control efforts. Malaria eradication remains a public health goal, so a better understanding of malaria transmission dynamics and population genetics is essential. Laiza and Myitsone are hot spots of *P. vivax* transmission along the China–Myanmar border [16]. Despite their proximity, we lack knowledge about the impact of control measures on parasite populations from these two areas. Thus, regional assessments will provide needed information on local epidemiological conditions to guide targeted malaria elimination efforts [26]. Here, we simultaneously characterized variations in *Pvmsp-3α* and *Pvmsp-3β* among *P. vivax* populations from two adjacent areas (Laiza and Myitsone) of Kachin State, Myanmar. The results showed different patterns and degrees of genetic diversity in the two populations. The higher genetic diversity of parasites from Myitsone suggested a higher level of malaria transmission there.

PCR–RFLP genotyping of *Pvmsp-3α* and *Pvmsp-3β* indicated high genetic diversity of the *P. vivax* population from this region, revealing large numbers of fragment-size alleles of the two polymorphic markers. Genotyping *Pvmsp-3α* also identified significantly different allele distributions and frequencies in the two neighboring areas, suggesting population differentiation. These two areas differed considerably in the prevalence of the *Pvmsp-3α* PCR size polymorphism, with the Laiza samples having more evenly distributed frequencies of the A, B, and C types as compared to the predominant A type found in the Myitsone samples (Fig. 3). Similarly, restriction digestions of the *Pvmsp-3α* amplicons corroborated this finding (Tables 2, 3). However, genotyping *Pvmsp-3β* was less informative and failed to differentiate these regional parasite populations, although *Pvmsp-3β* also had a large number of alleles (26 PCR–RFLP alleles).

A comparison of the parasite samples studied here with earlier publications suggested population changes in the China–Myanmar border, probably reflecting the impact of scaled-up control in the region. *Pvmsp-3α* Type A was the dominant infection type in central China, Thailand, Myanmar and other regions, while Type C was reportedly rare or absent [11]. The Myitsone samples more conformed to this allele type distribution. In Laiza, however, compared to the 2006–2008 parasite population, which showed that the Type A allele was most frequent, followed by Type B [14], this study identified a more evenly distributed *Pvmsp-3α* types, with Type C being the most abundant (37%). This difference may reflect recent parasite population expansion events revealed by whole-genome sequencing analysis [27], since the Laiza parasite population has experienced several outbreak events [16].

Multiple infections of genetically distinct clones of the same *Plasmodium* species are common in many places where malaria is endemic [29]. Some studies reported a reduced risk of clinical malaria in polyclonal infections [31], whereas other studies reported that mono-infections and prevalent genotypes were more likely to cause severe malaria than polyclonal infections [34]. Regardless, MOI may represent a measure to gauge the transmission intensity, with higher MOI occurring more often in high transmission areas. From our present study, mixed infections in Myitsone laborers appeared more frequent than in residents of Laiza, as estimated from *Pvmsp-3α* (*P* < 0.05). Consistently, a higher MOI was estimated from Myitsone laborers than residents of Laiza using *Pvmsp-3α* digested by *Hha* I (*P* < 0.05). Again, *Pvmsp-3α* provided more power to differentiate multiple infections than *Pvmsp-3β*. These results indicate greater malaria transmission intensity among these laborers during their stay in Myitsone.

We showed that PCR–RFLP analysis of *Pvmsp-3α* and *Pvmsp-3β* genes is a convenient method to identify the genetic diversity of the parasites from the China–Myanmar border [26]. Especially with *Pvmsp-3α*, it allowed us to differentiate parasite populations and identify mixed infections. However, PCR–RFLP analysis using these markers also has lower sensitivity in distinguishing closely related alleles, and may not be suitable for tracking cross-border parasite migration [36]. Deep sequencing of these amplicons would be more suitable for identifying the entire repertoire of these genetic variants [37].

## Conclusions

By PCR–RFLP genotyping *Pvmsp-3α* and *Pvmsp-3β* genes, we found evidence of genetic differences in *P. vivax* populations from two adjacent regions along the China–Myanmar border. Patients from Myitsone harbored more mixed infections and had higher MOI than those from Laiza.

## Supplementary Information

**Additional file 1.** Supplementary Tables.

**Additional file 2.** Supplementary Figures.
