# Supplementary material for: Genetic diversity of Plasmodium vivax populations from the China–Myanmar border identified by genotyping merozoite surface protein markers
Source: Trop Med Health. 2023 Jan 11;51:2. doi: 10.1186/s41182-022-00492-7 (PMC9832627; doi:10.1186/s41182-022-00492-7)
Supplement: Supplementary file 1 — Additional file 1. Supplementary Tables. [file 41182_2022_492_MOESM1_ESM.docx]

| **Supplementary Table 1** Primer sequences used for amplifying *Pvmsp-3α* and *Pvmsp-3β* genes | | | |
| --- | --- | --- | --- |
| Primers* | Sequence (5′-3′) ** | PCR cycling conditions | |
| *Pvmsp-3α* (P1) | F:5′-CAGCAGACACCATTTAAGG- 3' | 94°C 3 min/[94°C 30 s, 56°C 30 s, 68°C 2.5 min] × 35 cycles, 68°C 5 min |  |
| *Pvmsp-3α* (P2) | R:5′-CCGTTTGTTGATTAGTTGC- 3' |  |  |
| *Pvmsp-3α* (N1) | F:5′-GACCAGTGTGATACCATTAACC- 3' | 94°C 3 min/[94°C 30 s, 57°C 30 s, 68°C 2.5 min] × 30 cycles, 68°C 5 min |  |
| *Pvmsp-3α* (N2) | R:5′-ATACTGGTTCTTCGTCTTCAGG- 3' |  |  |
| *Pvmsp-3β* (P1) | F:5′-GTATTCTTCGCAACACTC- 3' | 94°C 3 min/[94°C 20 s, 54°C 20 s, 68°C 2.5 min] × 35 cycles, 68°C 5 min |  |
| *Pvmsp-3β* (P2) | R:5′^'^-CTTCTGATGTTATTTCCAG- 3' |  |  |
| *Pvmsp-3β* (N1) | F:5′-CGAGGGGCGAAATTGTAAACC- 3' | 94°C 3 min/[94°C 20 s, 54°C 20 s, 68°C 2.5 min] × 35 cycles, 68°C 5 min |  |
| *Pvmsp-3β* (N2) | R:5'-GCTGCTTCTTTTGCAAAGG- 3' |  | |

* P= Primary PCR; N = nested PCR

** F= Forward primer; R = Reverse primer.

**Supplementary Table 2** *Pvmsp-3*α *Alu* I PCR-RFLP allele types and the size of fragments in two *P. vivax* populations.

| Allele (PCR-RFLP) | The size of fragments (bp) |
| --- | --- |
| Type A | 1900-2000 |
| A1 | 500/250/200/150/100 |
| A2 | 500/300/200/150 |
| A3 | 500/400/200/150 |
| A4 | 500/250/200/150 |
| A5 | 500/200/150 |
| A6 | 500/300/200/100 |
| A7 | 500/400/250/200/150/100 |
| A8 | 500/350/200/150 |
| A9 | 500/250/200/150/100 |
| A10 | 500/250/150 |
| A11 | 500/300/250/150 |
| A12 | 500/400/250/150 |
| A13 | 500/200/150/100 |
| A14 | 500/350/250/170/150 |
| A15 | 500/400/350/200/150 |
| A16 | 50/350/200/150/100 |
| Type B | 1400-1500 |
| B1 | 500/250/200/150 |
| B2 | 500/250/150 |
| B3 | 500/350/200/150 |
| B4 | 500/200/170/150 |
| B5 | 500/300/200/100 |
| B6 | 550/200/150 |
| B7 | 500/350/200 |
| Type C | 1300-1100 |
| C1 | 500/250/150 |
| C2 | 500/400/250/200/150 |
| C3 | 500/400/200/150 |

**Supplementary Table 3** *Pvmsp-3α* *Hha* I PCR-RFLP allele types and the size of fragments in two *P. vivax* populations.

| Allele (PCR-RFLP) | The size of fragments (bp) |
| --- | --- |
| Type A | 1900-2000 |
| HA1 | 1000/450/250/200 |
| HA2 | 1000/500/350 |
| HA3 | 1000/400/250/200 |
| HA4 | 1000/250/200 |
| HA5 | 1000/400/300/250/200 |
| HA6 | 1000/300/250/200 |
| HA7 | 1000/500/250/150 |
| HA8 | 1000/500/450 |
| HA9 | 1000/250 |
| HA10 | 1000/350/200/150 |
| HA11 | 1000/500/350/200/150 |
| HA12 | 1000/400/200 |
| HA13 | 1000/500/400/250/200 |
| HA14 | 1000/500/300/250/200 |
| HA15 | 1000/500/400 |
| HA16 | 1000/450/350/250/150 |
| Type B | 1400-1500 |
| HB1 | 1000/400 |
| HB2 | 1000/350/150 |
| HB3 | 1000/500/250/200 |
| HB4 | 1000/500/400/250/200 |
| Type C | 1300-1100 |
| HC1 | 1000/200 |
| HC2 | 1000/400 |
| HC3 | 1000/250/200 |
| HC4 | 1000/400/250/200 |
| HC5 | 1000/150 |
| HC6 | 1000/500/200 |

**Supplementary Table 4** *Pvmsp-3β* *Pst* I PCR-RFLP allele types and the size of fragments in two *P. vivax* populations.

| Allele (PCR-RFLP) | The size of fragments (bp) |
| --- | --- |
| Type A | 1700-2200 |
| PA1 | 1000/800/600/300 |
| PA2 | 900/800/150 |
| PA3 | 1000/800/600/400/300/200/150 |
| PA4 | 1500/500/400/300/200 |
| PA5 | 1500/1000/350 |
| PA6 | 900/400/300/200 |
| PA7 | 800/600/300 |
| PA8 | 1500/900/600 |
| PA9 | 1500/800/400/300/200 |
| PA10 | 1500/800 |
| PA11 | 1250/800/300 |
| PA12 | 1500/1250/700/600 |
| PA13 | 1500/900/550/400 |
| PA14 | 1500/900/800 |
| Type B | 1400-1500 |
| PB1 | 1500/500/400/300/200 |
| PB2 | 800/600/550/500/400/300/200 |
| PB3 | 1500/1000/900/800 |
| PB4 | 800/600 |
| PB5 | 1500/1000/500 |
| PB6 | 1500 |
| PB7 | 1250/800/600 |
| PB8 | 1500/1000/600 |
| PB9 | 1250/1000/400 |
| PB10 | 1500/600 |
| PB11 | 800/700/150 |
| PB12 | 600/300 |
| PB13 | 1000/800/600/200 |
| Type D | 600-800 |
| PD | 700/150 |
