# Supplementary material for: Genetic diversity of Plasmodium vivax populations from the China–Myanmar border identified by genotyping merozoite surface protein markers
Source: Trop Med Health. 2023 Jan 11;51:2. doi: 10.1186/s41182-022-00492-7 (PMC9832627; doi:10.1186/s41182-022-00492-7)
Supplement: Supplementary file 2 — Additional file 2. Supplementary Figures. [file 41182_2022_492_MOESM2_ESM.pptx]

## Slide 1
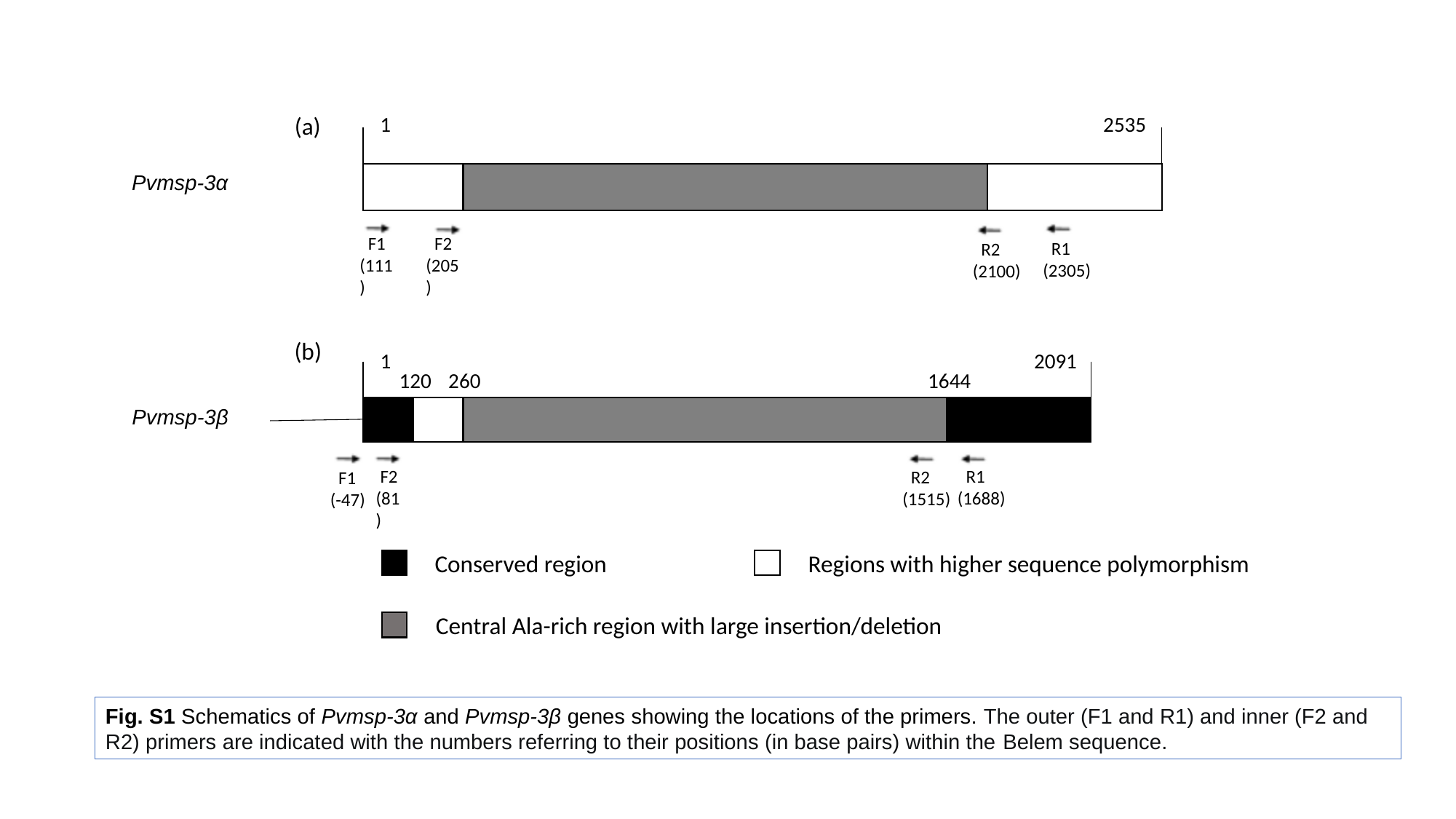

(a)
1
2535
Pvmsp-3α
 F1
(111)
 F2
(205)
 R1
(2305)
 R2
(2100)
(b)
1
2091
120
260
1644
Pvmsp-3β
 R1
(1688)
 F2
(81)
 R2
(1515)
 F1
(-47)
Conserved region
Regions with higher sequence polymorphism
Central Ala-rich region with large insertion/deletion
Fig. S1 Schematics of Pvmsp-3α and Pvmsp-3β genes showing the locations of the primers. The outer (F1 and R1) and inner (F2 and R2) primers are indicated with the numbers referring to their positions (in base pairs) within the Belem sequence.
